# Health and sustainable development; strengthening peri-operative care in low income countries to improve maternal and neonatal outcomes

**DOI:** 10.1186/s12978-018-0604-6

**Published:** 2018-10-05

**Authors:** Isabella Epiu, Josaphat Byamugisha, Andrew Kwikiriza, Meg Amy Autry

**Affiliations:** 1 NIH Fogarty Global Health Fellow, University of California Global Health Institute, San Francisco, CA USA; 2 Health Solutions International, P.O.Box 2336, Kampala, Uganda; 3 Makerere University, Kampala, Uganda; 4 Mbarara University, Mbarara, Uganda; 5 University of California San Francisco, CA, USA

**Keywords:** Maternal health, Universal health care, SDGs, Patient safety, Health systems, Health policy, Health financing, Global surgery, Safe anaesthesia

## Abstract

**Background:**

Uganda is far from meeting the sustainable development goals on maternal and neonatal mortality with a maternal mortality ratio of 383/100,000 live births, and 33% of the women gave birth by 18 years. The neonatal mortality ratio was 29/1000 live births and 96 stillbirths occur every day due to placental abruption, and/or eclampsia – preeclampsia and other unkown causes. These deaths could be reduced with access to timely safe surgery and safe anaesthesia if the Comprehensive Emergency Obstetric and Newborn Care services (CEmONC), and appropriate intensive care post operatively were implemented. A 2013 multi-national survey by Epiu et al. showed that, the Safe Surgical Checklist was not available for use at main referral hospitals in East Africa. We, therefore, set out to further assess 64 government and private hospitals in Uganda for the availability and usage of the WHO Checklists, and investigate the post-operative care of paturients; to advocate for CEmONC implementation in similarly burdened low income countries.

**Methods:**

The cross-sectional survey was conducted at 64 government and private hospitals in Uganda using preset questionnaires.

**Results:**

We surveyed 41% of all hospitals in Uganda: 100% of the government regional referral hospitals, 16% of government district hospitals and 33% of all private hospitals. Only 22/64 (34.38%: 95% CI = 23.56–47.09) used the WHO Safe Surgical Checklist. Additionally, only 6% of the government hospitals and 14% not-for profit hospitals had access to Intensive Care Unit (ICU) services for postoperative care compared to 57% of the private hospitals.

**Conclusions:**

There is urgent need to make WHO checklists available and operationalized. Strengthening peri-operative care in obstetrics would decrease maternal and neonatal morbidity and move closer to the goal of safe motherhood working towards Universal Health Care.

## Plain English summary

The 2015 Lancet Commission on Global Surgery reports that although remarkable gains have been made in global health in the past 25 years mortality and morbidity from common conditions needing surgery have grown in the world’s poorest regions. They estimate that 5 billion people lack access to safe, affordable surgical and anaesthetic care. Only 12% of the specialist surgical workforce practice in Africa and Asia, where a third of the world’s population lives. It is this inequality and the lack of access to safe surgical interventions in low- and middle-income countries (LMICs), that leads to unacceptable rates of morbidity and mortality.

Surgery and anaesthesia have long been neglected in global health work with the focus on communicable diseases. However, the recent World Health Assembly emphasized universal care for all, including strengthening of surgery and anaesthesia.

It is reported that 40% of maternal deaths could be averted with access to cesarean sections; therefore strengthening peri-operative surgical care in obstetrics would improve maternal and neonatal outcomes working toward the milestone of safe motherhood.

In conclusion, we believe that strengthening peri-operative care in obstetrics would decrease maternal and neonatal morbidity and move closer to the goal of safe motherhood working towards Universal Health Coverage.

## Background

Current estimates of 300,000 maternal deaths every year from pregnancy-related causes translates into a global maternal mortality ratio (MMR) of 216 deaths per 100,000 live births in 2015, a 37% reduction since 2000 [1]. Achieving the Sustainable Development Goals target of less than 70 maternal deaths per 100,000 live births by 2030 requires a global annual rate of reduction of at least 7.5%, more than double the rate achieved between 2000 and 2015 [1].

The potential for improvements in maternal and neonatal health outcomes with increased facility utilization in these countries is undermined by a lack of appropriate and timely treatment [2]. Providing antenatal care during pregnancy and skilled care during childbirth, as well as care and support in the weeks after childbirth can prevent most maternal and neonatal deaths [1]. As part of the continuum of care, safe surgery and safe anaesthesia are essential components of the comprehensive obstetric care package. Implementing timely and appropriate evidence-based practices during the peri-operative and immediate postoperative period can improve maternal and fetal health.

The main agenda in implementation of the Sustainable Development Goals by 2030 was to transform the world in 15 years building peaceful, inclusive societies as a foundation to free humanity from poverty, and ensure health and well being for all. Of the more than 130 million births occurring each year, there’s an estimated 2.6 million stillbirths, and another 2.7 million newborn death within the first 28 days of birth. The majority of these deaths occur in low-resource settings, often lacking skilled birth attendants. At least half of all stillbirths occurred in the intrapartum period [3]. In 2015, 1,700,000 babies were born in Uganda among young women aged 20–24, (33% gave birth by age 18) [4]. The Maternal Mortality in was 383/100,000 live births, with neonatal mortality of 29/1000 live births and 96 stillbirths occurring every day [5], due to placental abruption, and/or eclampsia – preeclampsia and other unkown causes. The major causes of maternal death are amenable to emergency surgical treatment, including hemorrhage (18%), [6] obstructed labor (8%) [6], unsafe abortion (18%) [6], in addition to uterine rupture, genital tract trauma, retained placenta, and ectopic pregnancy. These maternal and neonatal deaths could be prevented with access to timely safe surgery and safe anaesthesia by adequately implementing the Comprehensive Emergency Obstetric and Newborn Care services, and appropriate intensive care post operatively.

Anesthetic providers are also integral to the resuscitation and management of critically ill pregnant and postpartum women including sepsis and preeclampsia/eclampsia, responsible for 9% and 12% of maternal deaths, respectively [6].

In a study aimed at establishing the factors affecting maternal mortality in Uganda, investigating whether the number of antenatal Care visits, maternal education, age, area and region of residence had any effect on maternal mortality.

found mothers who make fewer or no ANC visits had a higher likelihood of death, both for themselves and their babies, than those who had more visits. It was also found that, mothers living in rural areas, compared to those in urban areas, are more likely to die due to pregnancy or related conditions [https://www.ajol.info/index.php/ajer/article/view/136040].

Acknowledging the complexity around childbirth an and the importance of surgical and anesthesia access in decreasing maternal and neonatal morbidity and mortality the, the World Health Organisation introduced the safe surgical and more recently the safe childbirth checklists [2, 7] as a means of improving the safety around the world; following reports that surgery may account for 40% of all hospital adverse events [8]. Furthermore, the World Health Organization (WHO) recommends that countries should achieve cesarean delivery (C-section) rates of 10% at a population level, to achieve reductions in maternal and newborn mortality rates [9].

Growing evidence has supported promotion of the use of checklists in several areas of healthcare with evidence of improved surgical outcomes [10–12]. In a multinational study involving eight hospitals from diverse economic settings, checklist implementation reduced death rate following surgery by approximately 50% [4]. All sites had a reduction in major postoperative complications, significantly at three sites, one in a high-income location and two in lower-income locations [4]. This is in-line with WHO pilot study that confirmed the conclusions of a number of earlier studies indicating that preoperative team introductions and briefings and postoperative debriefings contribute to improved surgical processes and outcomes [13].

Our group published data regarding availability of the WHO Safe Surgical Checklists in main referral hospitals throughout the East African region and reported 25% usage for obstetric patients [14]. Despite initial results demonstrating that properly implemented surgical checklists can make a substantial difference to patient safety; the implementation has not been straightforward. The reasons for this are varied and complex but include inconsistent leadership, lack of flexibility, and teamwork requirements [15].

The maternal mortality in Uganda is 343/ 100,000 live births [16] with 73% facility deliveries reported in the last 5 years [17]. Six percent of the live births were delivered by cesarean section with a cesarean section rate in urban areas at 11% and rural areas at 5% [18]; and there is currently no national policy regarding the use of the WHO checklist [14]. The WHO checklist was first introduced within the obstetric department at Mbarara Regional Referral Hospital in 2011 as part of a program to improve maternal care. This center has 15,000 deliveries per year. Here, checklist-training sessions were conducted over a five-month period by a visiting anaesthetist; but use of the checklist was not sustained after the volunteer left the hospital [15]. The main barriers identified were: the lack of clarity about responsibilities; lack of leadership and support from higher-level staff; no trained nurse to assist; and not enough time to complete the checklist [https://onlinelibrary.wiley.com/doi/full/10.1111/anae.13226].

We therefore sought to obtain data at the district level where most maternity care occurs, by assessing government and private hospitals in Uganda for the availability and usage of the WHO Safe Surgical Checklists and postoperative care of these patients.

## Methods

The cross-sectional survey was conducted in Uganda from September 2014 to August 2015. A total of 64 hospitals across Uganda were selected based on the criteria that they provided obstetric anaesthesia At least 15 hospitals from each region; East, West, North and Central were included:- 13 regional referral, 21 general (district), 7 private for profit and 7 private not for profit hospitals across the country. Our study represents 41% of all the hospitals in the country and 100% of the government regional referral hospitals, 15% of government general (district) hospitals and 33% of all private hospitals in Uganda. This included 26% of private for profit and 37% of the private non-for profit hospitals.

As published in by Epiu et al.** survey tool to evaluate compliance to the World Federation of Societies’ of Anaesthesiologists (WFSA) international guidelines for safe anaesthesia was developed [19]. These included quantitative and qualitative data on pre-operative assessment of patients, staffing and continuous monitoring intra-operatively and post-operatively. In this report we have included the peri-operative components on the use of the WHO Safe surgical Checklist, and documented the postoperative care of the parturient. I.E and trained research assistants interviewed on anaesthetist at each hospital, the hospital director and using a checklist assessed the theatre and recovery areas.

We purposefully selected all 14 regional referral hospitals as they are tertiary centres and serve as the surgical referral hospitals for lower health centres. We also randomly selected hospitals from other types of hospitals present in Uganda to include general (government district hospitals), private for profit and private not for profit hospitals.

With the help of a statistician, data was subsequently cleaned and coded into Epidata version 3.1. Range. Consistency and Validity checks were built in to minimize errors. Data was exported and analyzed using STATA version 14 (Statcorp, College Station, Texas, USA).

Ethical approval was obtained from Makerere University School of Medicine Research and Ethics Committee (SOMREC) REC REF No 2014–133, the appropriate participating hospitals, and the Uganda National Council for Science and Technology. Informed consent was obtained from all individuals participating in the study.

## Results

**Fig. 1:**
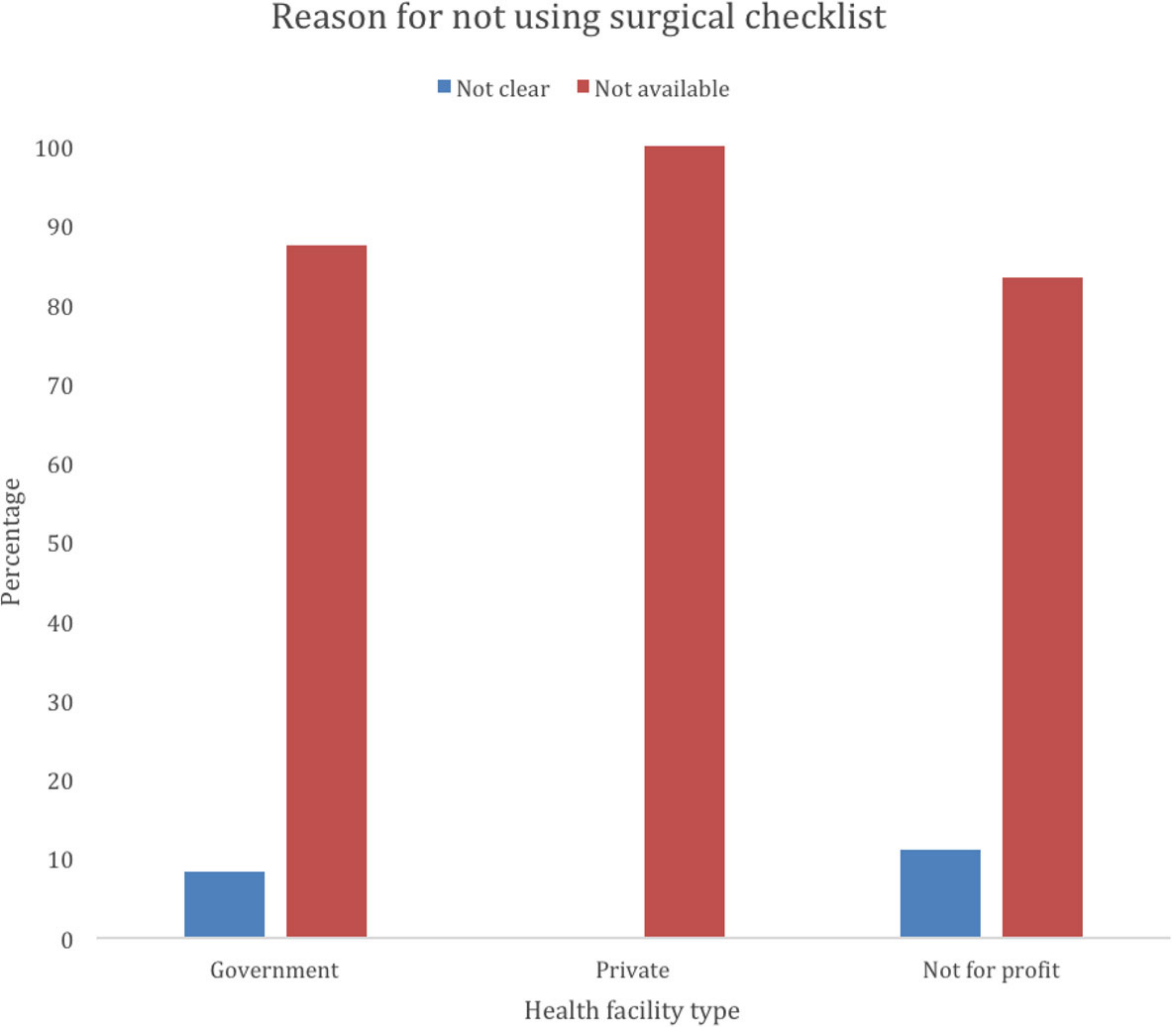
Reason why WHO safe surgical checklist was not used at the 64 Hospitals in Uganda. Note: *p*-value = 0.989 (No differences in proportions among the health facilities – meaning that almost all facilities didn’t use the surgical check list because they were not available)

Only 22/64 (34.38%: 95% CI = 23.56–47.09) used the WHO Safe surgical checklist, and Table 1 highlights its per facility. The difference between the WHO Checklist usage in government and private hospitals was not statistically significant (*p* = 0.081) Lack of availability of the checklist was the reason given for why the WHO Safe surgical checklist was not used, Fig. 1.

**Table 1:** Use of the WHO Safe surgical checklist intraoperative, post operative care and monitoring, and availability of ICU Services

Variable	Health facility type N (%)			Chi square *p*-value
	Government *N* = 34	Private *N* = 7	Not for profit *N* = 23	
Use of surgical checklist				
Yes	11 (32.35)	5 (71.43)	6 (26.09)	
No	23 (67.65)	2 (28.57)	17 (73.91)	0.081
Patients taken care of postoperatively in recovery				
Yes	9 (29.03)	5 (71.43)	6 (27.27)	
No	22 (70.97)	2 (28.57)	16 (72.73)	0.075
Postoperative pain review				
24 h	30 (88.24)	5 (71.43)	21 (95.45)	
48 h	3 (8.82)	1 (14.29)	1 (4.55)	
> 48 h	1 (2.94)	1 (14.29)	0	0.345
Basic monitoring ICU services available				
Yes	2 (6.06)^a^	4 (57.14)	3 (13.64)^a^	
No	31 (93.94)	3 (42.86)	19 (86.36)	0.002

^a^missing one

Post operatively the patients were cared for in a recovery area in 20/64 (33%) of the hospitals. Although 88% stated that they ensured the patients were pain free; only 7.8% of the anesthetists revisited the patients 48 h later for pain review. Only 9/64 of the health facilities (14%, *P*-Value 0.002) had intensive care unit services available for postoperative care of critical parturients requiring close monitoring, Table 1. Of these, access to ICU services for parturients post-operatively were mainly available in private hospitals (57%), Table 1.

Most private hospitals had an organized post anaesthesia care unit sometimes with a dedicated member of staff monitoring the patients, compared to improvised spaces or corridors in the other hospitals. Additionally, only 6% of the government hospitals and 14% not-for profit hospitals had access to ICU services for postoperative care compared to 57% of the private hospitals.

## Discussion

Safe anaesthesia and safe surgery are indispensable components of emergency comprehensive obstetric healthcare. To address the complexities around childbirth the WHO has introduced the safe surgical and safe childbirth checklists. This cross-sectional survey at 64 hospitals provided data on peri-operative use of the WHO Safe Surgical Checklist and postoperative care for the obstetric patients in Uganda.

Seventy-one percent of the private hospitals used the safe surgical checklist, which demonstrates a disparity in delivery of care between the rich who can pay out of pocket, insured patients and poor patients who cannot afford the high quality services. The use of the safe surgical checklist in government hospitals was 32%, which can possibly be attributed to lack of resources and/or shortage of personnel for implementation. Generally there was no significant difference in proportions among the health facilities – meaning that almost all facilities didn’t use the surgical checklist because they were not available. The response “It is not available” is perhaps a reflection of the culture and attitudes in these hospitals rather than an indication of the actual inavailability, similar to our published data from the multi-national East Africa survey [14]. This response may reflect a lack of enthusiasm for checklist use because it can actually be accessed online. However, future research may be required on motivations for use and access to internet in various settings. In addition, more training may be needed with providers on where to locate the checklist online. Other challenges of access, like availability to internet can be addressed by regularly providing printed copies to surgical centers.

From our study private facilities also provided more postoperative care compared to the government and not for profit hospitals. An ICU requires an intensivist and specialized ICU nursing team, which is a big challenge in a country that has very few specialists relative to the growing population. A 7-year retrospective audit from 2003 to 2009 by Kwizera et al. estimated that there were 33 ICU beds in the whole country for a population of 33 million people in Uganda, representing 0.1 ICU beds/100,000 population. Head injuries were a common reason for ICU admission and associated with the highest mortality rates in this audit and 23/33 ICU beds in the whole country were at private hospitals [18]. This limitation is further compounded by a well-documented dearth of anaesthesiologists- a critical human resource for intensive care units [20, 21].

The private hospitals provide ICU service, potentially requiring catastrophic out of pocket financial expenses for those who do not have insurance. Uganda has a large population that is below the poverty line and most patients cannot afford the expense of a private hospitals resulting in an overwhelming number of patients in the government hospital which is then compounded by other factors like understaffing eventually affecting the quality of services given to patients. This data presents significant barriers to achieving Universal Health Care particularly in accessing safe surgery and safe anaesthesia, an essential component of the comprehensive emergency obstetric and neonatal care. Similarly, Wall et al. state that intrapartum-related neonatal deaths can be substantially reduced by improving the quality of services for all childbirths that occur in health facilities, identifying and addressing the missed opportunities to provide effective interventions to those who seek facility-based care [22].

Bringing about a change in health policy on a worldwide basis by guaranteeing health and well-being for all age groups – the crux of the Sustainable Development Goal, is easier said than done. Strategic health financing is one the key aspects that can push this objective forward, along with considerable efforts by the global health workforce. If measures are to be properly exercised regarding global health, around 18 million extra health workers are needed by 2030.

Data form 2005 to 2015 suggests that more than 40% of countries have less than one physician per 1000 people and around half have fewer than three nurses or midwives per 1000 people. If the situation isn’t rectified, the circumstances might become worse due to an increased rate of migration of qualified health professionals to high income countries. Without targeted interventions, the situation in some of these countries could be further exacerbated by increased labour migration of trained health personnel towards high-income countries with greater demand and compensation, thereby further undermining already vulnerable health systems [23].

It isn’t all bad news though as the Lancet Commission on surgery reports that quality of health care has improved globally in the last 25 years, but the progress is inconsistent. Unfortunately morbidity and morality from common condition requiring surgery have spiked in the poorest of places. Almost 5 billion are yet to be provided access to affordable and safe anesthesia and surgical care when required. Steps should be undertaken to remove the disparity of access in certain regions as well [24]. Sub-Saharan Africa, North Africa and Middle East have a population of 1305 million with a total surgical burden of 73 million Cases per 100,000 population, there is a total estimated unmet need of 38,871,266 surgical cases [25, 26]. In addition, within those countries, as our study demonstrates, there is a disparity of access to evidence based interventions with poor women suffering greater burden.

To address this gap in surgery, the Lancet Commission recommends a work force density of 20 Surgeons Obstetricians and Anaesthetists per 100,000 population, with 5 per 100,000 population as anaesthetists in each country. Our research demonstrated the profound lack of safe anaesthesia in East Africa with delays in performing cesarean deliveries when needed due to lack of anaesthetists, lack of equipment and emergency drugs, shortage of blood supply, and a failed referral system. In a previous publication, we found that for a population of 142.9 million in the East African Community, there were only 237 anesthesiologists, with a workforce density of 0.08 in Uganda, 0.39 in Kenya, 0.05 in Tanzania, 0.13 in Rwanda, and 0.02 in Burundi per 100,000 population in each country [27].

To achieve the sustainable development goals of 2030, it is imperative to operationalize more medical schools and professional colleges in the region and train more doctors equipping them with essential skills in surgery and anaesthesia. We must implement the evidence based international guidelines for safer outcomes including WHO Checklists.

While The Lancet Commission focuses on increasing surgical volume; in low to middle income countries the value of these surgical procedures needs to be closely considered. Highly cost effective procedures that are affordable for the community should be implemented.

There are many larger discussions to be had regarding health care in sub-Saharan Africa to include infrastructure building to improve roads and transportation for quicker access to critical surgical procedures. Healthcare financing is also an essential discussion where millions of families are one disease away from poverty following catastrophic out of pocket medical payments. The shortage of ICU services in government facilities forces patients to pay out of pocket in private facilities. It is time we develop modeled health approaches to bridge the gap in domestic health financing in low and middle-income countries. If communities are able to pay for clean pumped and/or piped water, and electricity, how much more to contribute to a collective pool for better health services?

## Conclusions

Surgery and anaesthesia have long been neglected in global health work with the focus on communicable diseases. However, the recent World Health Assembly emphasized universal care for all, including strengthening of surgery and anaesthesia. Increasing access to quality cesarean sections and obstetric safe anaesthesia would improve maternal and neonatal outcomes working toward the milestone of safe motherhood. Now is the time to re-configure the global health target to specifically address these health disparities in low-income countries; developing health leadership, mentorship, strengthening health systems, designing and implementing local and international policies in order to achieve equity in health care.

## Abbreviations

ICU: Intensive Care Unit
LMICs: Low-and middle-income countries
MMR: Maternal mortality ratio
WHO: World Health Organisation
